# Outcomes Among Patients With or Without Obesity and With Cancer Following Treatment With Immune Checkpoint Blockade

**DOI:** 10.1001/jamanetworkopen.2022.0448

**Published:** 2022-02-28

**Authors:** Seong-Keun Yoo, Diego Chowell, Cristina Valero, Luc G. T. Morris, Timothy A. Chan

**Affiliations:** 1 Center for Immunotherapy and Precision Immuno-Oncology, Cleveland Clinic, Cleveland, Ohio; 2 The Precision Immunology Institute, Icahn School of Medicine at Mount Sinai, New York, New York; 3 Department of Surgery, Memorial Sloan Kettering Cancer Center, New York, New York

## Abstract

This cohort study assesses the association between pretreatment body mass index in patients with 16 cancer types and immune checkpoint blockade treatment outcomes.

## Introduction

Obesity is a risk factor for cancer, but it may also be associated with improved treatment outcomes in patients with cancer.^1^ This notable phenomenon is called the *obesity paradox*. The benefits of obesity in patients treated with immune checkpoint blockade (ICB)^2,3^ and the underlying mechanisms^2^ are being examined but the scale and spectrum of benefit across cancer types is unknown. To address this gap in knowledge, we evaluated pretreatment body mass index (BMI) in 1840 patients across 16 cancer types.

## Methods

This study was approved by the institutional review board of Memorial Sloan Kettering Cancer Center and followed the Strengthening the Reporting of Observational Studies in Epidemiology (STROBE) reporting guideline for cohort studies. Patients who received at least 1 dose of ICB treatment from 2014 through 2019 were included. Data were analyzed from January 2014 to December 2021. All patients provided written informed consent for tumor sequencing. BMI was measured within 30 days before treatment. We calculated BMI as weight in kilograms divided by height in meters squared. Normal weight (BMI, 18.5-24.9), overweight (BMI, 25-29.9), and obesity (BMI, ≥30) were defined based on the World Health Organization guidelines. All patient tumors were sequenced using Memorial Sloan Kettering-Integrated Mutation Profiling of Actionable Cancer Targets (MSK-IMPACT). We defined tumor mutational burden (TMB) as the total number of somatic nonsynonymous mutations per megabase. Detailed patient characteristics are described in the eMethods in the Supplement.

All statistical tests were performed with R programming language, version 4 (R Foundation for Statistical Computing). Kaplan-Meier plot and Cox proportional hazards regression analyses were performed with the survminer package. *P* < .05 was considered significant.

## Results

Of a total of 1840 patients, the median patient age was 63.84 years (IQR, 55.66-71.16 years). Of that total, 1059 patients (57.55%) were male.

**Figure 1.**
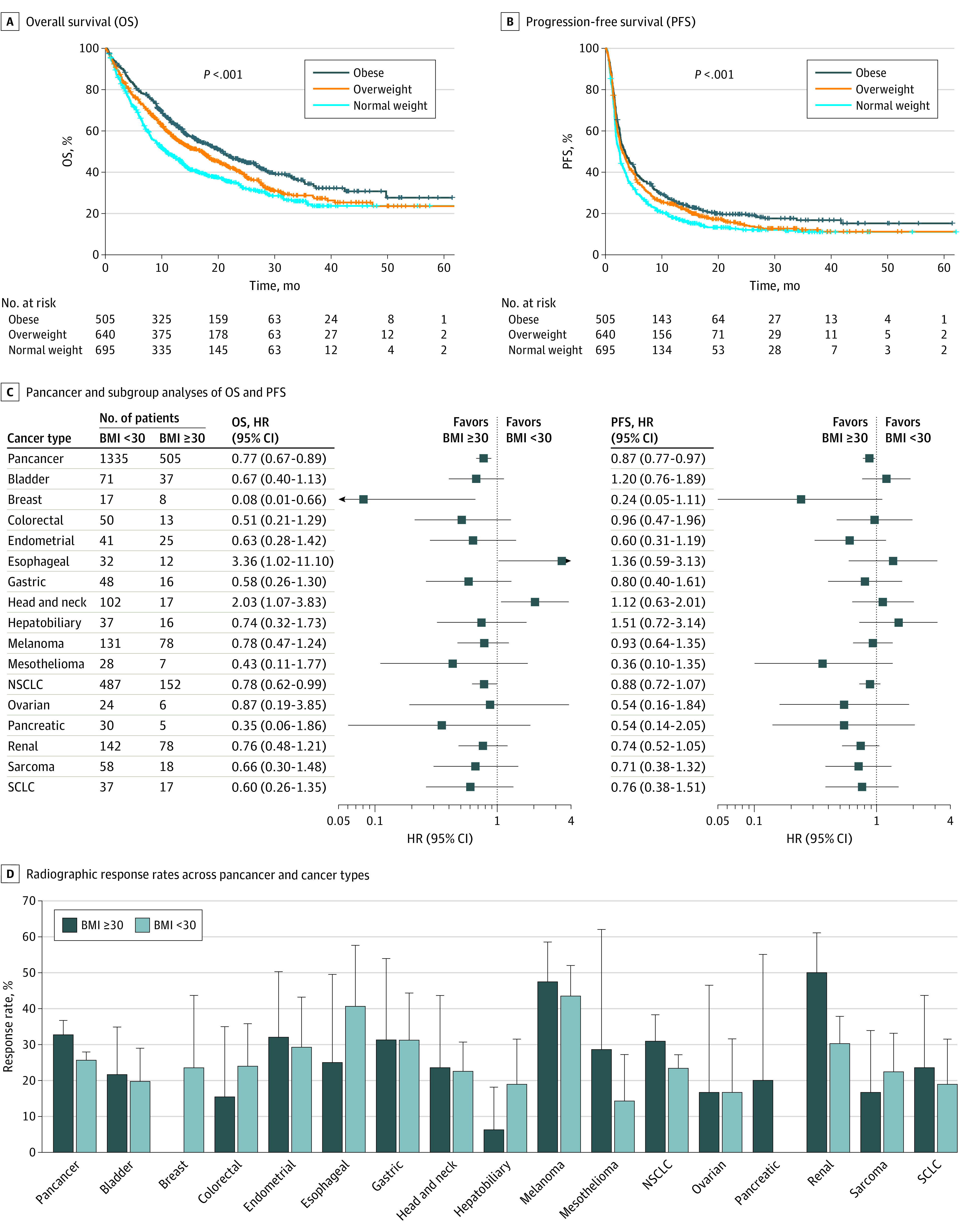
Association of Obesity With Survival and Radiographic Response in Patients Following Immune Checkpoint Blockade (ICB) Treatment All hazard ratios and 95% CIs were adjusted for tumor mutational burden, age, sex, tumor stage, drug class, neutrophil-to-lymphocyte ratio, ICB line of treatment, and Eastern Cooperative Oncology Group performance score with the Cox proportional-hazards regression model. For pancancer analyses, cancer type (melanoma, non–small cell lung cancer [NSCLC], and others) was adjusted. SCLC indicates small cell lung cancer. Radiographic response rates along with the 95% Wald CIs according to BMI groups across pancancer and 16 cancer types (D).

In pancancer survival analyses, patients with obesity showed better overall survival (OS) after ICB treatment than patients with overweight (hazard ratio [HR], 0.82; 95% CI, 0.70-0.96) or normal weight (HR, 0.67; 95% CI, 0.57-0.78). Patients with overweight showed better OS after ICB treatment than patients with normal weight (HR, 0.81; 95% CI, 0.71-0.93). Patients with obesity showed better progression-free survival (PFS) after ICB treatment than patients with overweight (HR, 0.89; 95% CI, 0.78-1.02) or normal weight (HR, 0.77; 95% CI, 0.68-0.87), and patients with overweight showed better PFS after ICB treatment than patients with normal weight (HR, 0.86; 95% CI, 0.76-0.97) (Figure 1, A and B). Multivariable Cox regression analyses showed having BMI of 30 or higher was associated with better survival than having BMI less than 30 independent of known factors associated with ICB treatment outcomes, and we found this association across many cancer types (Figure 1, C).

We then assessed the association between obesity and radiographic response rate. In a pancancer analysis, having obesity was associated with higher response rates compared with having normal weight or overweight (32.67% [165 of 505] vs 25.62% [342 of 1335]) (OR, 0.71; 95% CI, 0.56-0.89) (*P* = .003) (Figure 1D). The association of obesity with response to ICB was not uniform across cancer types. The largest response rate difference by BMI groups was observed in kidney cell carcinoma (50.00% [39 of 78] vs 30.28% [43 of 142]) (OR, 0.44; 95% CI, 0.24-0.80) (*P* = .01).

**Figure 2.**
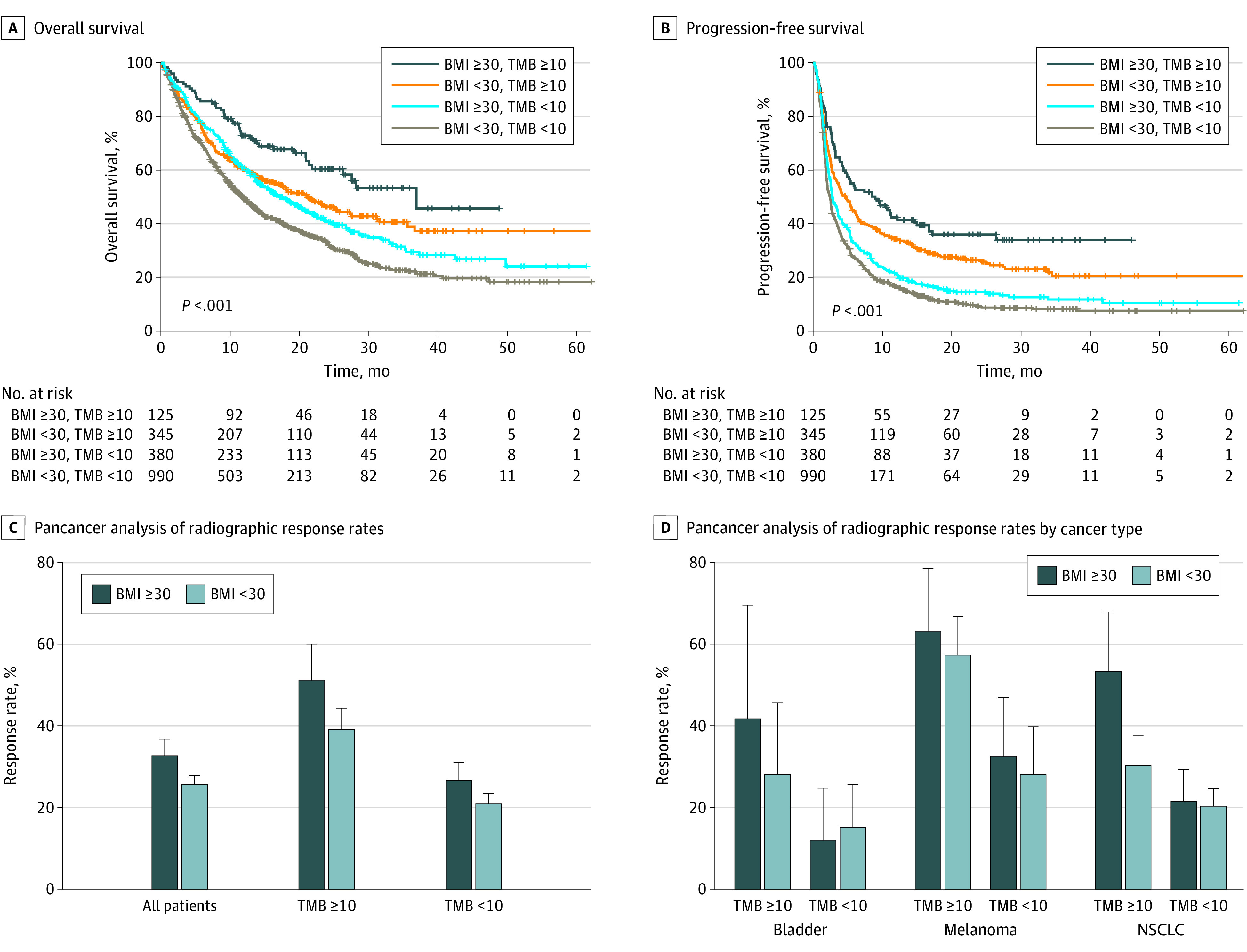
Combined Associations of Obesity and Tumor Mutational Burden (TMB) With Patient Outcome Pancancer analyses of (A) OS and (B) PFS of 4 groups stratified by body mass index (BMI) and TMB. All hazard ratios and 95% CIs were calculated by univariate analysis. *P* values were generated by log-rank test. Pancancer analysis of radiographic response rates with the 95% Wald CIs according to the BMI and TMB categories (C). ORs and 95% CIs for each comparison are presented. Response rates with the 95% Wald CIs according to the BMI and TMB groups across the selected cancer types (D). Cancer types with more than 10 patients in each group were selected.

We further stratified patient groups using BMI along with TMB, which is associated with response to ICB in many cancer types.^4^ The high TMB group was defined by the cutoff of greater than 10 mutations per megabase, which was approved by the FDA as a biomarker for anti–programmed cell death 1.^5^ Patients with BMI of 30 or higher had better OS after ICB treatment than patients with BMI of less than 30 in the TMB greater than 10 group (HR, 0.64; 95% CI, 0.47-0.89) and in the TMB less than 10 group (HR, 0.74; 95% CI, 0.64-0.85) (Figure 2, A). Furthermore, patients with BMI of 30 or higher showed better PFS after ICB treatment than patients with BMI of less than 30 in the TMB greater than 10 group (HR, 0.75; 95% CI, 0.58-0.97) and in the TMB less than 10 group (HR, 0.84; 95% CI, 0.74-0.95) (Figure 2, B).

Response rate difference by BMI group was larger in the TMB of 10 or more group (51.20% [64 of 125] vs 39.13% [135 of 345]) (OR, 0.61; 95% CI, 0.40-0.95) (*P* = .03) than the TMB less than 10 group (26.58% [101 of 380] vs 20.91% [207 of 990]) (OR, 0.73; 95% CI, 0.55-0.97) (*P* = .03) (Figure 2, C). This association was also found in a subgroup analysis (Figure 2, D). In patients with non–small cell lung cancer, the group with BMI of 30 or higher and TMB of 10 or higher showed significantly higher response rate than the group with BMI of less than 30 and TMB of 10 or higher (53.33% [24 of 45] vs 30.26% [68 of 335]) (OR, 0.38; 95% CI, 0.18-0.79) (*P* = .008), however there was no difference in response rate between the BMI groups with TMB less than 10 (21.50% [23 of 107] vs 20.30% [68 of 335]) (OR, 0.93; 95% CI, 0.53-1.66) (*P* = .90).

## Discussion

The results of this cohort study suggest that obesity is associated with immunotherapy outcomes in patients with certain cancer types. A limitation of the study was that statistical power in subgroup analyses was small due to sample sizes and cancer-type-specific confounding variables such as the International Metastatic RCC Database Consortium risk score were not addressed in this study. Therefore, the results in some cancer types warrant further study with larger cohorts to address specific confounding variables in more detail. In addition, BMI is not a perfect assessment of obesity because it cannot distinguish muscle from body fat. Hence, body fat measurement methods such as dual-energy x-ray absorptiometry may have value in future studies.^6^

## Notes

Supplement. **eMethods.** Patient Characteristics, Outcomes, and Statistics
eReferences
