# Supplementary material for: Outcomes Among Patients With or Without Obesity and With Cancer Following Treatment With Immune Checkpoint Blockade
Source: JAMA Netw Open. 2022 Feb 28;5(2):e220448. doi: 10.1001/jamanetworkopen.2022.0448 (PMC8886536; doi:10.1001/jamanetworkopen.2022.0448)
Supplement: Supplement. — eMethods. Patient Characteristics, Outcomes, and Statistics eReferences [file jamanetwopen-e220448-s001.pdf]

Supplemental Online Content

Yoo SK, Chowell D, Valero C, Morris LGT, Chan TA. Outcomes among patients with or without obesity and with cancer following treatment with immune checkpoint blockade. *JAMA Netw Open.* 2022;5(2):e220448. doi:10.1001/jamanetworkopen.2022.0448

eMethods. Patient Characteristics, Outcomes, and Statistics

eReferences

This supplemental material has been provided by the authors to give readers additional information about their work.

© 2022 Yoo SK et al. *JAMA Network Open*.

eMethods

Patient characteristics

This study includes 1,840 patients across 16 cancer types diagnosed from 2014 through 2019 with a history of only one solid tumor type. All patients received at least one dose of immune checkpoint blockade (ICB) treatment, excluding neoadjuvant or adjuvant treatment. Patients were grouped into three body mass index (BMI) categories based on the World Health Organization guidelines: normal weight (18.5≤BMI<25), overweight (18.5≤BMI<25), and obese (BMI≥30). In this cohort, 27.45% of patients were normal weight (695/1,840), 34.78% were overweight (640/1,840), and 37.77% were obese (505/1,840).

Patient characteristics of the normal weight group were as follows: median age (interquartile range [IQR]) 62.99 (54.26-71.39) years; gender 360 females, 335 males; cancer type 10 breast, 13 mesothelioma, 15 ovarian, 18 pancreatic, 19 esophageal, 20 endometrial, 20 hepatobiliary, 20 small-cell lung cancer (SCLC), 25 gastric, 32 bladder, 32 colorectal, 32 sarcoma, 49 melanoma, 66 head & neck, 68 renal, 256 non-small cell lung cancer (NSCLC); tumor stage 36 I-III, 648 IV, 11 unknown; ICB response 158 responder, 537 non-responder; ICB line of treatment 225 first line, 470 subsequent line; drug class 4 anti-CTLA4, 598 anti-PD1/PD-L1, 93 combo; Eastern Cooperative Oncology Group (ECOG) performance status 208 0-grade, 386 1-grade, 49 2-grade, 6 3-grade, 1 4-grade, 45 unknown; median tumor mutational burden (TMB; IQR) 5.3 (2.6-9.8) mut/Mb; median neutrophil-to-lymphocyte (NLR) ratio (IQR) 4.64 (2.97-7.86).

Patient characteristics of the overweight group were as follows: median age (IQR) 64.60 (57.07-71.87) years; gender 220 females, 420 males; cancer type 7 breast, 15 mesothelioma, 9 ovarian, 12 pancreatic, 13 esophageal, 21 endometrial, 17 hepatobiliary, 17 SCLC, 23 gastric, 39 bladder, 18 colorectal, 26 sarcoma, 82 melanoma, 36 head & neck, 74 renal, 231 NSCLC; tumor stage 34 I-III, 601 IV, 5 unknown; ICB response 184 responder, 456 non-responder; ICB line of treatment 237 first line, 403 subsequent line; drug class 1 anti-CTLA4, 531 anti-PD1/PD-L1, 108

© 2022 Yoo SK et al. *JAMA Network Open*.

combo; ECOG performance status 260 0-grade, 288 1-grade, 34 2-grade, 8 3-grade, 2 4-grade, 48 unknown; median TMB (IQR) 6.1 (3.3-10.5) mut/Mb; median NLR ratio (IQR) 4.37 (2.75-7.41).

Patient characteristics of obese group were as follows: median age (IQR) 63.46 (56.10-70.61) years; gender 201 females, 304 males; cancer type; 8 breast, 7 mesothelioma, 6 ovarian, 5 pancreatic, 12 esophageal, 25 endometrial, 16 hepatobiliary, 17 SCLC, 16 gastric, 37 bladder, 13 colorectal, 18 sarcoma, 78 melanoma, 17 head & neck, 78 renal, 152 NSCLC; tumor stage 34 I-III, 601 IV, 5 unknown; ICB response 165 responder, 340 non-responder; ICB line of treatment 178 first line, 327 subsequent line; drug class 1 anti-CTLA4, 400 anti-PD1/PD-L1, 104 combo; ECOG performance status 212 0-grade, 231 1-grade, 27 2-grade, 4 3-grade, 1 4-grade, 30 unknown; median TMB (IQR) 5.3 (3.0-9.8) mut/Mb; median NLR ratio (IQR) 3.8 (2.56-6.40).

Outcomes

We examined patient outcomes as described in the previous study[1]. Overall survival (OS), progression-free survival (PFS), and radiographic response to ICB were evaluated. From the first infusion of ICB to any cause of death was defined as OS. When patients received multiple doses of ICB, we calculated OS based on the day of first dose. PFS was calculated from the first infusion of ICB to disease progression or any cause of death; patients without progression were censored at their last date of attended appointment at the Memorial Sloan Kettering Cancer Center. We measured response to ICB based on RECIST v1.1 criteria[2]. When formal RECIST evaluation was not available, we manually reviewed physician's notes and imaging reports to classify overall best response using the same criteria based on change in the sum of diameters of target lesions. We defined responders as patients with complete response or partial response, and non-responders as patients with stable disease or progressive disease.

Statistics

© 2022 Yoo SK et al. *JAMA Network Open*.

All statistical tests were performed with R programming language (https://www.r-project.org/). Kaplan-Meier plot and Cox proportional hazards regression analyses were performed with the survminer package (https://CRAN.R-project.org/package=survminer).

© 2022 Yoo SK et al. *JAMA Network Open*.

eReferences

1. Valero C, Lee M, Hoen D, et al. Pretreatment neutrophil-to-lymphocyte ratio and mutational burden as biomarkers of tumor response to immune checkpoint inhibitors. *Nat Commun.* 2021;12(1):729.

2. Eisenhauer EA, Therasse P, Bogaerts J, et al. New response evaluation criteria in solid tumours: revised RECIST guideline (version 1.1). *Eur J Cancer.* 2009;45(2):228-247.

© 2022 Yoo SK et al. *JAMA Network Open*.
